# Low FODMAP diet in children and adolescents with functional bowel disorder: A clinical case note review

**DOI:** 10.1002/jgh3.12231

**Published:** 2019-08-02

**Authors:** Stephanie C Brown, Kevin Whelan, Richard B Gearry, Andrew S Day

**Affiliations:** ^1^ Department of Paediatrics University of Otago Christchurch Christchurch New Zealand; ^2^ Department of Nutritional Sciences Kings College London London UK; ^3^ Department of Gastroenterology Christchurch Hospital Christchurch New Zealand; ^4^ Department of Medicine University of Otago Christchurch Christchurch New Zealand; ^5^ Department of Paediatrics Christchurch Hospital Christchurch New Zealand

**Keywords:** dietetics, functional bowel disorder, gastroenterology, nutrition, pediatrics

## Abstract

**Background and Aims:**

Functional bowel disorders (FBD), such as irritable bowel syndrome (IBS), are increasingly more common in children and affect up to 20% of children. The etiology is multifactorial with no clear organic cause. Symptoms are recurrent and are associated with a reduced quality of life, school absences, and psychological challenges. Treatment options are variable. FODMAPs are short‐chained carbohydrates, poorly absorbed by the gastrointestinal tract due to their increased osmotic activity and excess gas production from the bacterial fermentation process. There is a paucity of data examining dietary interventions that restrict carbohydrates in children with IBS. The aim of this study was to examine the use of the low FODMAP diet (LFD) in children with an FBD.

**Methods:**

A retrospective clinical case note review of children with an FBD managed with an LFD was undertaken. Anthropometry and clinical data were collected by a pediatric gastroenterology dietitian. An IBS satisfaction survey was used to assess diet outcomes. Statistical analyses were completed using Excel.

**Results:**

Of the 29 children included in this study, complete resolution of gastrointestinal symptoms was observed for 11 of 12 (92%) of those with bloating, 13 of 15 (87%) of those with diarrhea, and 17 of 22 (77%) of those with abdominal pain. Twenty‐three (79%) participants reported an improvement of symptoms. Fructans were the most common symptom‐causing carbohydrate.

**Conclusion:**

The LFD is a useful dietary treatment strategy for children with FBD. This study adds to the small body of evidence supporting FODMAP dietary interventions in children with FBD. Further prospective studies are required.

## Graphical abstract

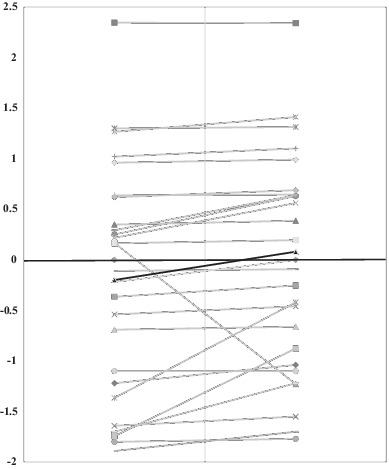

Functional bowel disorders (FBD) in children are becoming increasingly more common where there are data. The low FODMAP (LFD) diet is used as a successful dietary therapy for treating irritable bowel syndrome in adults; however, a paucity of data exists examining the use of this diet in children. This retrospective clinical case note review showed that the LFD is a useful dietary treatment strategy in children and adolescents with FBD and adds to the small body of evidence supporting LFD interventions in children.

## Introduction

Functional bowel disorders (FBD) affect 20% of children worldwide.^1^, ^2^ Their etiology involves a combination of genetic, environmental, and psychological factors, with a clear organic cause not yet identified.^3^ Children with FBD have recurrent and variable symptoms that may persist into adulthood^4^ and are associated with a reduced quality of life, school absences, and psychological challenges.^5^, ^6^ Due to the heterogeneity of FBD, a single treatment strategy is often unsuccessful in completely eliminating symptoms.^7^

Irritable bowel syndrome (IBS) is a common FBD. Parents of children with IBS report that specific foods may trigger their child's symptoms,^8^ and more than 90% of adolescents with IBS make changes to their diet.^6^ In adults with IBS, a low FODMAP diet (LFD) is well‐established as a safe and effective dietary strategy to alleviate symptoms.^9^, ^10^

FODMAP is an acronym for fermentable oligosaccharides (fructans and galacto‐oligosaccharides [GOSs]), disaccharides (lactose), monosaccharides (fructose), and polyols (e.g. sorbitol, mannitol and xylitol). Individual FODMAPs can increase osmotic activity and, therefore, increase water secretion in the small intestinal lumen (e.g. mannitol, fructose), and when they are fermented by the gut microbiome, they stimulate colonic gas production (e.g. fructans and GOSs).^10^, ^11^ IBS symptoms are associated with FODMAP consumption in people with hypersensitivity to these alterations in small intestinal water and colonic gas.

There is currently a paucity of data examining the efficacy and safety of dietary interventions that restrict carbohydrates in children with IBS.^9^ Given the increase in FBD in children worldwide, studies are required to determine the efficacy and safety of the LFD in children. While a limited number of studies have shown that the LFD reduces FBD symptoms in children, these have been short‐term efficacy trials utilizing tightly controlled feeding study designs,^8^, ^12^, ^13^, ^14^ and therefore, the effectiveness of the LFD in free‐living individuals advised to follow the diet in routine clinical practice has not been demonstrated. The aim of this study was to describe our experience with LFD in managing symptoms in children with FBD.

## Methods

### *Participants*

A retrospective chart review study of children with an FBD managed with an LFD was undertaken. Children who had been referred for dietetic assessment and consideration of an LFD between January 2015 and February 2018 at one pediatric center (Christchurch Public Hospital, New Zealand) were identified. Inclusion criteria were children aged between 4 and 17 years, presenting with chronic, persistent, and relapsing symptoms consistent with an FBD (NICE Criteria),^15^ along with the exclusion of organic causes (such as coeliac disease and food allergy) by a pediatric gastroenterologist and a request from the referring clinician to commence an LFD. Patient stress and/or anxiety was determined from either the child's historical medical notes or by the referring gastroenterologist who addressed this in the referral letter to the dietitian.

### *Protocol*

All children and their families received three dietetic consultations in line with best practice recommendations.^16^ Children were seen for their first dietetic consult within 3 weeks of the gastroenterologist's referral, for their second consult between 3 and 4 weeks after the commencement of the restriction phase, and for their last consult after they completed the reintroduction phase. At each consultation, a standardized series of questions were asked to determine the frequency and severity of symptoms, habitual dietary intake and food preferences, and efficacy of the LFD and to explore specific carbohydrate intolerance(s) during the reintroduction phase. Dietary resources used throughout this study were developed using the Monash University, Australia adult FODMAP resources adapted for pediatrics by a pediatric gastroenterology dietitian. The meal plan used throughout this study was created to reflect a typical New Zealand child's lunch box in alignment with the Ministry of Health's Healthy Eating Guidelines for Children and Young People.^17^

At the initial dietetic consultation, the families provided prerecorded 3‐day food and beverage diaries. Weight and height were measured and recorded by the same dietitian, and the child's dietary intake, nutritional adequacy, food preferences, and consumption of high FODMAP foods were assessed qualitatively. FBD symptoms were previously recorded by the pediatrician at the time of the child's last medical review. The dietitian completed the appropriate dietary education using a standardized resource, including a meal plan. Children were asked to complete the 4‐week restriction phase as strictly as possible and to maintain a food, beverage, and symptom diary throughout the duration of the 4 weeks.

At the second dietetic consult, weight and height was remeasured. The effectiveness of the FODMAP restriction phase was determined by the symptoms recorded, and in children who had experienced symptom resolution, education was provided regarding how to complete the reintroduction phase to establish any carbohydrate intolerance(s). One individual FODMAP carbohydrate was reintroduced each week over a period of 3 days using preselected foods and serving sizes in alignment with the Monash resources. Any established carbohydrate intolerance and its associated symptoms were documented by the child or parent/guardian using his or her symptom diary. Children who did not experience any symptom relief were advised to liberalize their diet at this time.

At the final dietetic consult, outcomes were discussed, and an individualized dietary plan was developed considering any established carbohydrate intolerance(s). Families were advised to retest any established FODMAP trigger every 3 months from the initial testing period using the same process as in the reintroduction phase to assess any change in tolerance. Resolution of symptoms was determined by asking participants explicit questions, including whether they experienced partial, complete, or no change in symptoms.

### *Anthropometry*

Weight and height were measured at the baseline and the second consultation using the same scales and stadiometer. Weight was measured using a bariatric scale suitable for up to 360 kg (SECA, New Zealand), and height was measured using SECA 264, a wall‐mounted stadiometer (SECA, New Zealand), in light indoor clothes; body mass index (BMI) was calculated. “Over‐nutrition” was classified as BMI above 85% for age, and “undernutrition” was classified as BMI less than 15% for age.^18^

### *Gastrointestinal symptom response*

Gastrointestinal (GI) symptom response was assessed for clinical purposes using subjective measures, including physician documentation in clinical notes based on the NICE guidelines,^15^ standardized questions asked by the dietitian at the time of dietary consults, and symptom diaries. Carbohydrate intolerances were determined subjectively during the reintroduction phase of the LFD based on symptom exacerbation during challenges. Any gut symptoms that were triggered by consuming a predetermined quantity of FODMAP were recorded by the participant in his or her symptom diary as the presence or absence of symptoms and by asking the participant for a detailed description of symptoms experienced in the food diary comments section.

### *Outcome measures: Retrospective survey*

The effectiveness of the intervention was measured by performing a symptom response survey via telephone between 2 and 28 months after the child's third [final] dietetic consult.^19^ This survey measured symptom outcomes using a questionnaire containing eight questions. The patients rated symptom changes for bloating, abdominal pain/discomfort, flatulence, diarrhea, constipation, nausea, and energy levels using a 7‐point Likert scale taken from the validated IBS Global Improvement Scale (substantially worse, moderately worse, slightly worse, no change, slightly improved, moderately improved, substantially improved, or never had the symptom).^20^

A further four statements relating to satisfaction with symptom response and the LFD were also included. These statements were scored using a 5‐point Likert scale (strongly disagree, disagree, neutral, agree, and strongly agree). All data were anonymous and confidential. Patients were encouraged to answer questions honestly to reduce response bias. Survey data were collected via telephone.

### *Statistical analysis*

Mean (standard deviation [SD]) and number (percentage) were calculated for continuous and categorical data, including baseline characteristics, anthropometry, GI symptoms, and outcomes. Differences for symptom response and satisfaction using both subjective outcomes pre‐ and post‐LFD completion and the retrospective telephone survey were calculated.

Data from the retrospective telephone survey were collapsed to provide clinically meaningful data and robust data distribution. Symptom data were collapsed into dichotomous responses as “improved” (slightly improved, moderately improved, substantially improved) or “not improved” (no change, slightly worse, moderately worse, substantially worse). Data were also collapsed to describe the magnitude of improvement: “worsened/no change” (no change, slightly worse, moderately worse, substantially worse), “slightly improved,” “moderately improved,” and “substantially improved.” Satisfaction responses were collapsed into agree and disagree responses, and a composite score was calculated to determine overall subjective diet efficacy.

Ethical approval was granted by the University of Otago Ethics Committee (Health) HD18/026.

## Results

### *Participants and baseline characteristics*

Twenty‐nine children were included in this retrospective case note review, 27 of whom completed the LFD as described (Table 1). Two 15‐year‐old females voluntarily withdrew from the restriction phase after 2 weeks due to self‐reported lack of effect. All 29 patients referred for dietetic input attended their consult, and all 29 patients were included in the clinical analyses where data were available. They comprised 20 females and nine males. Two females were classified as “over‐nutrition.” The majority of participants experienced a combination of lower GI symptoms, with 22 (76%) children reporting abdominal pain and 12 (41%) reporting abdominal distention. Upper GI symptoms, including nausea and esophageal reflux, were also experienced by two (6.8%) and three (10%) children, respectively.

**Table 1:** Baseline characteristics of 29 children with functional bowel disorders managed with a low FODMAP diet

	Females (*n* = 20)	Males (*n* = 9)	Total (*n* = 29)
Characteristics			
Age, years, mean (SD)	10.9 (4)	13.5 (3.23)	11.7 (3.94)
Age, median (IQR)	11.5 (7.5)	14.4 (4.9)	12.6 (0.5)
Age, years, min–max	4.7–17.6	8–17.9	4.7–17.9
Clinical diagnosis of stress and/or anxiety, *n* (%)	6 (30)	1 (11)	7 (24)
Ethnicity, *n* (%)			
New Zealand European	18 (62)	8 (28)	26 (90)
European (other)	2 (7)	1 (3)	3 (10)
Body mass index†			
Underweight	0 (0)	0 (0)	0 (0)
Normal weight	18 (90)	9 (100)	2 (93)
Overnutrition	2 (10)	0 (0)	2 (7)
Presence of functional bowel disorder symptoms, *n* (%)			
Abdominal pain	19 (95)	3 (33)	22 (76)
Distention	9 (45)	3 (33)	12 (41)
Diarrhea	4 (20)	4 (44)	8 (27)
Constipation	6 (30)	0 (0)	6 (21)
Alternating stools	4 (20)	3 (33)	7 (24)
Poor intake	3 (15)	2 (22)	5 (17)
Nausea	1 (5)	1 (11)	2 (7)
Esophageal reflux	1 (5)	2 (22)	3 (10)

† BMI classification was determined using the Z‐scores of the World Health Organization (WHO) growth reference age of 5–19 years.

### *Survey outcome measuring the response to FODMAP restriction*

Symptom responses from the retrospective survey are shown in Table 2. Of the 29 children included in this study, complete resolution of GI symptoms was observed in 92% of those with bloating, 87% of those with diarrhea, and 77% of those with abdominal pain (Table 2). Only one of the seven participants with stress and/or anxiety reported complete resolution of their symptoms, whereas four reported a partial resolution. Constipation only was completely resolved in three of eight children, while three children with constipation reported a partial resolution. Most participants reported a “substantial improvement” of their FBD symptoms. Those with abdominal bloating had the highest rate of improvement, followed by those with abdominal pain. Of the children, 80% reported increased energy levels when assessed by the retrospective symptom survey.

**Table 2:** Symptom response to the low FODMAP diet obtained during the retrospective survey 2–28 months after completion of the diet

Symptom	Symptom total *n* (%)†	No change or worse	Slightly improved	Moderately improved	Substantially improved	Improved‡ *N* (%)
Abdominal pain	22 (78)	5 (23)	2 (9)	2 (9)	13 (59)	17 (77)
Bloating	12 (41)	1 (8)	1 (8)	2 (17)	8 (67)	11 (92)
Diarrhea	15 (52)	2 (13)	2 (13)	1 (7)	10 (67)	13 (87)
Constipation	13 (45)	2 (15)	3 (23)	1 (7)	7 (54)	11 (85)
Nausea/reflux	5 (17)	1 (20)	2 (40)	1 (20)	1 (20)	4 (80)
Energy levels	10 (34)	2 (29)	3 (30)	1 (10)	4 (40)	8 (80)
Composite score§	29 (100)	13 (45)	14 (48)	9 (31)	47 (160)	23 (79)

† Total number of children experiencing symptoms in each category.

‡ 79% of children experienced some improvement of symptoms, dichotomized as improved (slightly, moderately, or substantially) or not improved.

§ Total outcome scores for symptoms experienced: Those with symptoms of alternating stools were included in both the diarrhea and constipation categories.

Nausea and reflux were combined.

### *Pattern of carbohydrate intolerance*

During the reintroduction phase, several participants experienced symptoms to more than one carbohydrate as follows: fructans was the most common intolerance (18, 67%), followed by lactose (16, 56%), polyols (2, 7%), fructose (2, 7%), and GOSs (2, 7%). Six (24%) children specifically identified that apples (fructose and sorbitol) triggered symptoms.

### *Anthropometry*

**Figure 1:**
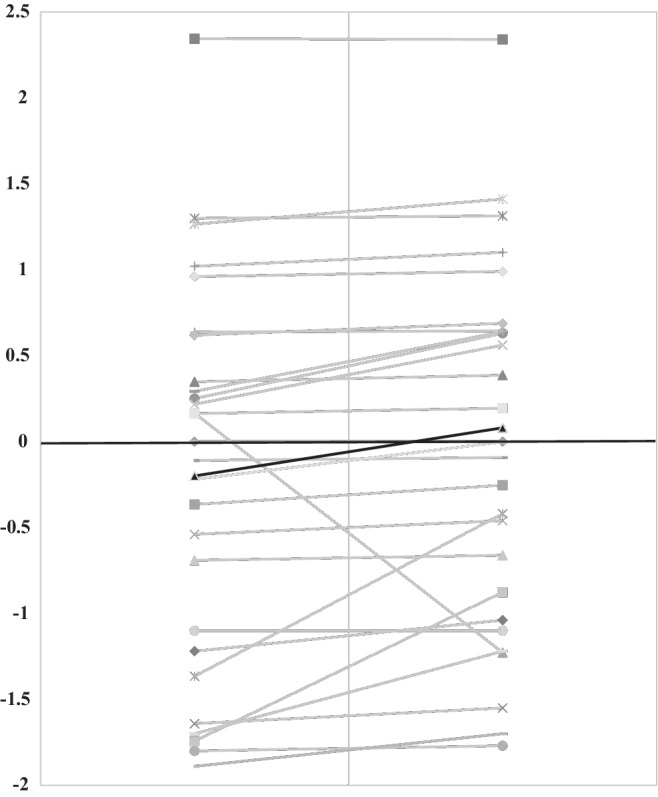
Changes in participants' *Z*‐scores for weight at baseline and after completing the low FODMAP diet. The *Z*‐score for weight at baseline and after following the low FODMAP diet in 29 children with functional bowel disorders. The gray lines indicate individual patient data, and the black line indicates the mean values in the population (Baseline‐0.2, Post‐LFD 0.08).

From the time of the child's initial dietetic consult to their second consult, children gained, on average, 617 g ranging from a 2.7 kg weight loss to a 6.3 kg weight gain. These weight differences did not affect total mean BMI z‐score (Fig. 1).

### *Age‐related outcomes*

Children aged 10 years and younger experienced a greater percentage of complete resolution of symptoms (5, 56%) compared to those aged 10 and older (9, 45%). Both age groups reported almost equivalent episodes of partial resolution of symptoms (≤10 years, 3 (33%) *vs* ≥10 years, (6, 30%)). No change in symptoms were reported by 11% (1) of those aged 10 years and under and 25% (5) of those aged 10 years and older.

### *Dietary satisfaction survey*

The participant response for dietary satisfaction is reported in Table 3. Eighteen (62%) children reported that they found the written information/resources provided easy to understand, and 23 (79%) also found the diet easy to follow. Nine (31%) children were not satisfied with the improvement of their overall symptoms, and 17 (59%) were not interested in changing their diet further to improve symptoms.

**Table 3:** Dietary satisfaction response from retrospective survey, *N* (%)

*n* (%)	Strongly disagree	Disagree	Neutral	Agree	Strongly agree
I found the written information easy to understand	0 (0)	0 (0)	2 (7)	18 (62)	9 (31)
I found the diet easy to follow (both restriction and reintroduction phases)	0 (0)	2 (7)	2 (7)	23 (79)	2 (7)
Overall I am satisfied with the improvement in my symptoms	0 (0)	9 (31)	4 (14)	10 (34)	6 (21)
I would be interested in further changing my diet to improve symptoms	0 (0)	17 (59)	7 (24)	5 (17)	0 (0)

## Discussion

This retrospective study evaluated the effect of the LFD on symptoms and anthropometry in free‐living children with an FBD. This study showed that more than 50% of children with an FBD who complete the LFD restriction and reintroduction process will have complete resolution of symptoms, particularly those with lower GI symptoms. Furthermore, only 6 of the 29 children in the current study did not respond to the dietary intervention. These results also illustrate that the use of the LFD in these children did not lead to any significant detrimental impact on body weight in the short term.

These results are similar to those previously reported in adults. The LFD appears to be more efficacious for those with symptoms of flatulence, diarrhea, abdominal pain, and distention.^9^ Moreover, the carbohydrate intolerances identified during the reintroduction process are similar for both adults and children, with fructan and lactose being the most common.^9^ Similar to the current study, adult studies indicate that episodes of anxiety exacerbate GI symptoms and that more females are affected by FBD than males.^10^

The majority of children in this study agreed that the written dietary resources were easy to understand and that the LFD was easy to follow, which is clinically useful given that there are currently no international LFD guidelines for pediatrics. Interestingly, nine children were not satisfied with their response to the LFD, which may reflect either a poor response to or poor tolerance of the LFD. This is important to discuss with children and their families prior to commencing the LFD so that expectations of the expected response rates are realistic.

In children, data on the use of LFD as a treatment strategy for FBD are relatively lacking. Researchers from the United States have performed a number of clinical trials on LFD in pediatric patients with IBS.^2^, ^8^, ^14^, ^21^ These studies have shown a rapid symptom reduction within 2 days, which is substantially faster than adult studies.^19^ The present study did not assess the timeline of LFD impact on symptoms; however, this would be useful to test in future prospective studies as this will help to establish clearer pediatric guidelines. Although the present study was retrospective, it showed the LFD to be an appropriate treatment for children with FBD in a real‐world setting. These data are unique but complementary to those demonstrated in shorter studies by other groups.^2^, ^8^, ^14^, ^21^

The increased rates of anxiety in patients with FBD is consistent with other studies that show high rates of anxiety‐related disorders in pediatric patients with FBD.^22^, ^23^ Given that 17% of children in this study experienced stress and anxiety, psychological strategies need to be used in concert with dietary interventions. Randomized and observational studies provide relatively compelling but limited evidence that psychological interventions are associated with improved pain tolerance, reduced anxiety, increased nonpain behavior, and improved self‐management.^24^, ^25^, ^26^, ^27^, ^28^, ^29^, ^30^, ^31^, ^32^, ^33^, ^34^, ^35^, ^36^, ^37^, ^38^, ^39^ In a prospective study in which 322 children with functional abdominal pain were followed to young adulthood, the lifetime risk of anxiety and depression were 51 and 40%, respectively (compared 20 and 16%, respectively, in controls).^40^ The explanation of a biopsychosocial model of functional disease can be helpful as it frames the disease in terms of being a positive diagnosis rather than a diagnosis of exclusion. The family's ability to accept such a biopsychosocial model of pain may be an important factor in the child's recovery.^41^

Thoughtful consideration should be given prior to commencing any restriction diet in pediatrics to ensure that nutritional status is not compromised. However, the LFD permits children to consume foods from each of the core food groups, therefore minimizing nutritional inadequacies when appropriately implemented with advice from a specialist dietitian.^42^ One particular study to carefully measure the effect of dietary advice for FODMAP restriction on habitual dietary intake found no difference in micronutrient intake compared with controls, except for a lower calcium intake,^43^ presumably a result of a lower intake of dairy foods. A reduced calcium intake in children in the long term would adversely impact bone health, which is critical in this time of rapid growth and development. A diet low in FODMAPs leads to low concentrations of Bifidobacteria and higher concentrations of Roseburia and Ruminococcus in the gut microbiota,^44^, ^45^, ^46^ and the long‐term repercussions of this change are unknown, particularly in children.

Caution should therefore be exercised in ensuring that the FODMAP restriction phase is not continued for long periods and that FODMAPs are reintroduced into the diet to test for tolerance in order to mitigate impacts on nutritional status and gut microbiome diversity. It is essential that dietary restrictions in children (including the LFD) are overseen by specialist dietitians to ensure nutritional adequacy. One child in this clinical case note review experienced a considerable weight loss of 6.3 kg during the study despite dietetic input. This was, in part, a result of a participant's history of anxiety and associated decreased appetite and intake, for which she had previously had psychological input. Moreover, two children gained a mild amount of weight post‐LFD. These mild gains were, in part, a reflection of GI symptom relief and the subsequent ability of these children to eat more food than when they were feeling unwell. This is a commonly observed occurrence as GI symptoms can affect appetite and oral intake.

The current study has numerous limitations. First, the study was conducted retrospectively with a review of patient charts to obtain key patient data. Second, the study comprised a relatively small sample size, but the sample represents the totality of patients at this specialist center over 3 years. A 16‐point IBS satisfaction survey was utilized in the current study to measure symptoms (bloating, pain, alternating stools, etc.); however, this was completed retrospectively and, in some instances, more than 2 years after the child received his or her initial dietetic consult. The population was unbalanced for gender (20 females, nine males); however, this reflects the female predominance in FBD prevalence in children.^47^ Finally, there was no control group in this particular study, which would have provided more robust comparative data and potentially enabled blinding of the intervention and mitigation of the placebo effect.

As a strength, the current study featured consistency of care provided to participants, with the same pediatric gastroenterologist and the dietitian throughout the duration of the study. Dietary guidelines also remained consistent using comprehensive data on food composition. To our knowledge, the present study is the only one to report efficacy and safety data for LFD in free‐living pediatric patients with FBD in a real‐world setting. These important data support the need for a prospective study to understand the efficacy and safety of the LFD in children in more detail.

## Conclusion

In conclusion, the LFD is a useful dietary treatment strategy in children and adolescents with FBD. The current study adds to the small body of evidence supporting LFD interventions in children. Further prospective studies are needed in children to better define factors that contribute to the sensitivity of FODMAPs and to evaluate the impacts on nutritional status and growth and the gut microbiome and the consequences of long‐term dietary restriction.
